# Vitamin D levels in a population from Argentina with metabolic disorders

**DOI:** 10.1097/j.pbj.0000000000000159

**Published:** 2022-06-17

**Authors:** Maria Constanza Luciardi, Mariano Nicolás Alemán, Daniela Martinez, Mirta Centeno Maxzud, Analía Soria, Mirta Ester Aldonati, Hector Lucas Luciardi

**Affiliations:** a Cátedra de Práctica Profesional, Departamento de Bioquímica Aplicada, Facultad de Bioquímica, Universidad Nacional de Tucumán,; b Hospital Centro de Salud Zenón J. Santillán,; c Centro de Endocrinología Diabetes y Nutrición,; d Cátedra Patología Molecular. Facultad de Bioquímica, Química y Farmacia,; e Tercera Cátedra de Patología y Clnica Médica, Facultad de Medicina, Universidad Nacional de Tucumán, Tucumán, Argentina.

**Keywords:** excess weight, hypothyroidism, type 2 diabetes, vitamin D

## Abstract

Metabolic disorders are a public health problem worldwide. The vitamin D status in patients with metabolic diseases is not a routine procedure. The aim of this study was to determine the prevalence of vitamin D deficiency and examine the correlation between vitamin D status and cardiometabolic parameters in Latin American population with metabolic disorders.

**Methods:**

This observational study with a cross-sectional design included 151 patients with metabolic disorders (type 2 diabetes, hypothyroidism, type 2 diabetes with hypothyroidism, and excess weight). A fasting blood sample was collected and analyzed to determine the levels of 25-hydroxyvitamin D, calcium, glucose, hemoglobin A1c, thyroid-stimulating hormone, and free thyroxine. Anthropometric and blood pressure measurements were also performed.

**Results:**

According to vitamin D values established by the Institute of Medicine, subjects with metabolic disorders group showed: 23% risk to bone health (9.42 ±3.O4ng/mL), 45% risk of insufficiency/deficiency (17.05 ±2.12ng/mL), and 32% had sufficient levels (26.34±6.74ng/mL), whereas healthy subjects group showed significantly higher values than metabolic diseases group (37.25± 7.72). In addition, vitamin D levels were inversely correlated with elevated body mass index (29.13±5.15kg/m^2^), systolic blood pressure (126.50± 15.60 mm Hg), fast blood glucose (106.29±33.80 mg/dL), and hemoglobin A1c (6.40% ± 1.38%) values.

**Conclusion:**

Subjects with metabolic disorders and with adequate nutritional intake of vitamin D-rich foods and frequent exposure to sunlight have low serum vitamin D concentrations compared to the general population and vitamin D status should be assessed in these patients.

## Introduction

Pancreas and thyroid diseases are the most common endocrine and metabolic disorders in the adult population, such as obesity, diabetes, and hypothyroidism.^1^ Obesity is a chronic, epidemic, preventable, high-cost illness, characterized by the excessive or abnormal accumulation of fat in the adipose tissue that carries health risks and implications. According to the World Health Organization, overweight and obesity are defined as body mass index (BMI) values ≥25 and 30kg/m^2^, respectively, considered as excess weight (EW) patients.^2^

Type 2 diabetes (T2D) is a complex disorder that depends on genetic and environmental factors. It is a metabolic disease characterized by the presence of chronic hyperglycemia, which results from insufficient insulin secretion or *decrease of its biological action* in different tissues *like* skeletal muscle, liver or adipose tissue. T2D represents a health problem, especially in low-income countries where the mortality by this condition is 80%.^3,4^

Hypothyroidism is a common endocrinopathy with metabolic manifestations caused by thyroid hormone deficiency. Primary or clinical hypothyroidism occurs with elevated concentrations of thyroid-stimulating hormone (TSH) and low free thyroxine (free T4) levels.^5^ Subclinical hypothyroidism is characterized by high serum TSH levels with normal free T4 and free triiodothyronine (T3).^6^

Vitamin *is key in the hormonal regulation of bone mineral homeostasis.* Vitamin D2 (ergocalciferol) and D3 (cholecalciferol) are obtained through the intake of vitamin D2- and D3-rich foods (fish and its oils, eggs, yeasts, and fortified foods) or photosynthesis in the skin through *sun exposure. But these vitamins do not have biological activity and need to be activated through 2 hydroxylation reactions: in the liver producing 25-hydroxy metabolites and in the kidneys producing calcitriol.*^7^

There is no agreed definition of vitamin D deficiency; however, most agencies internationally suggest that a serum 25-OH vitamin D (25-OHD) <12ng/mL (30nmol/L) represents a risk to bone health. Furthermore, according to vitamin D recommendations, serum 25-OHD concentrations between 12 and 20 ng/ mL (30–50nmol/L) should indicate a risk of insufficiency/ deficiency for some members of the population and levels above 20ng/mL (50nmol/L) should consider sufficiency for almost everyone.^8,9^ Vitamin D deficiency is a public health problem worldwide present in almost all communities, even those predicted to have high levels of vitamin D.^10^ Given the absence of uniformly accepted definitions, previous reviews have reported substantial variations in the prevalence of vitamin D deficiency among countries worldwide, with estimates ranging from 2% to 90% depending on the cut-off value and study population selected.^11^ In Argentina, the situation is more complicated, as limited information did not allow meta-analyses to be carried out. From 1987 to 2015,10 studies reported the prevalence of vitamin D deficiency in our country and only one of them corresponds to the Northern region.^12^ Vitamin D deficiency is concerning because it has been shown to be independently associated with a higher risk of mortality from different causes.^13^ Indeed, it is commonly linked to musculoskeletal diseases, such as childhood rickets, adult osteomalacia and osteoporosis.^14^ In addition, in recent decades it has been associated with chronic diseases such as obesity, T2D, endocrinopathies with metabolic manifestations and inflammatory diseases.^15^ Vitamin D determination in these patients has not gained the needed recognition in Latin America.

Recent evidence of vitamin D actions and its relationship to metabolic disorders shows the importance of keeping it’s values within the normal range. Nevertheless, there is insufficient information in our region about vitamin D levels in these patients. In this context, the aim of this cross-sectional study was to determine the prevalence of vitamin D deficiency and examine the correlation between vitamin D status and cardiometabolic parameters in Latin American population with metabolic disorders.

## Methods

### Study design/patient selection

Observational study with a cross-sectional design included 151 patients with metabolic disorders: T2D, primary hypothyroidism, T2D with primary hypothyroidism (T2D + hypothyroidism), EW, (41 men/110 women), and 22 healthy subjects (HS, 8 men/ 14 women) registered in the Center of Endocrinology, Diabetes, and Nutrition (CEDyN) *from Northern Argentina (state of Tucuman; 26°49’26.9” S 65°13.35’1” O) from March 2018 through March 2019. The patient’s mean age was 57.0± 12.0 and 56.0± 6.0years in the metabolic disorder and HS groups, respectively.*

Patients with T2D were *being* treated with oral hypoglycemic drugs such as metformin or in combination therapy with short-and long-acting insulin. Patients with hypothyroidism were being treated with thyroid hormone replacement therapy. *Patients with arterial hypertension were being treated* with pharmacological and nonpharmacological approaches to achieve target blood pressure (BP) range.

#### Inclusion criteria

Residents with frequent sun exposure and dietary calcium and vitamin D intake (evaluated by a structured questionnaire focusing on specific foodstuffs naturally containing vitamin D), similar economic status and educational level. All patients were at least 5 years since diagnosis of the disease and underwent a full clinical evaluation, including weight, height, BMI, waist circumference (WC), BP, and family history of diabetes and/or thyroid diseases. Arterial hypertension was defined as BP ≥130/85mm Hg and/or current use of antihypertensive drugs (values according to the cutoffs established by International Diabetes Federation and the American Heart Association/National Heart, Lung, and Blood Institute).^16^

#### Exclusion criteria

Patients taking drugs known to affect vitamin D metabolism (supplements, glucocorticoids, or anticonvulsants) or with infectious diseases, renal and hepatic diseases, acute process, malabsorption disorder, or other endocrine diseases.

#### Anthropometric and blood pressure measurements

Weight and height were measured by mechanical adult scale (Rome BPP-S w/Altimeter, Hijos de Francisco DINO S.R.L., Rosario, Argentina), with light clothing and no shoes, with ankles together, relaxed shoulders and both arms at the sides of the bodies. BMI was estimated using Quetelet index (weight/size^2^). WC was measured with an anthropometric tape measure (Lufkin W606PM, New York, NY) at the site of maximum circumference midway between the lower ribs and the anterior superior iliac spine. BP was averaged from 2 seated measurements using an automatic monitor (Omron Healthcare Co., HEM-7120, China).

### Biochemical parameters

Blood samples were obtained between 8 and 10 AM,by venipuncture, after night time fasting. Glycemic control was evaluated by hemoglobin A1c (HbA1c) (immunoturbidimetric inhibition method, Wiener Lab, Argentina), fasting blood glucose (FBG) (enzymatic method, Wiener Lab, Argentina) using Mind-ray BS-380 chemistry analyzer (Mindray Bio-Medical Electronics CO., Ltd., Shenzhen, China) and capillary blood glucose levels. TSH, Free T4 and 25-OHD levels were determined by chemiluminescence in Mindray CL-1000i chemistry analyzer (Mindray Bio-Medical Electronics CO., Ltd). Calcium values were measured by colorimetric method in Mindray BS-380 chemistry analyzer.

Levels of 25-OHD were used to classify patients into: risk to bone health (<12ng/mL), risk of insufficiency/deficiency (12-20 ng/mL), and sufficiency (20ng/mL).^9^

### Statistical analysis

Statistical analysis was performed using the IBM SPSS Statistics, ver. 25.0 (IBM Co., Armonk, NY). Kolmogorov-Smirnov test was used to determine quantitative variables distribution. All data were expressed as frequency and percentage for categorical data and mean ± standard deviation. Differences in study participants’ characteristics were compared across subgroups with chi-square test for categorical variables and *T* test as appropriate. Differences in mean levels of continuous variables between subgroups were determined using one-way analysis of variance with Tukey post-hoc test as appropriate for multiple comparisons. Pearson correlation coefficient was used to investigate correlations between parameters. A *P* value <0.05 was considered significant.

### Ethical statement

Study approved by Tucuman University CEI ethics committee (Tucumán, Argentina) (Ethical Committee No. 7/2017) (October 25, 2017); written informed consent for participation was obtained from all the patients.

## Results

Clinical characteristics of patients with metabolic disorders and HS are shown in Table 1. Significant differences were found in anthropometric measurements (weight, height, BMI, and WC); systolic blood pressure (SBP), FBG, HbA1c, and 25-OHD. There were no significant differences in the rest of the parameters evaluated.

**Table 1:** Clinical and biochemical characteristics of the groups studied.

	Metabolic diseases	Healthy Subjects	P
N	151	22	—
Male/Female	41/110	8/14	.376
Age (yr)	59 (47–67)	57 (51-62)	.297
Weight (kg)	77.69 ± 16.4	68.29±8.44	.010
Height (m)	1.63 ± 0.08	1.69±0.09	.001
BMI (kg/m^2^)	29.13±5.15	23.72±0.75	.001
WC male (cm)	99±16.56	93.75±5.67	.038
Female (cm)	88.39±14.79	78.43±6.11	.038
SBP (mm Hg)	126.50±15.60	116.82±4.78	.002
DBP (mm Hg)	68.46±8.95	70.00±6.90	.256
FBG (mg/dL)	106.29± 33.80	84.00±6.65	.001
HbA1c (%)	6.40±1.38	5.25±0.31	.001
TSH (uUI/mL)	2.13±1.36	1.82±0.59	.696
Free-T4 (ng/dL)	1.78±1.73	1.51 ± 0.57	.460
Ca (mg/dL)	8.98±0.65	9.11 ± 0.66	.230
25-OHD (ng/mL)	18.90±7.61	37.24±7.72	.001

All data are expressed as frequency and percentage for categorical data and mean ± standard deviation. Significant *P<* .05.

25-OHD=25-OH vitamin D, BMI = body mass index, Ca=calcium, DBP=diastolic blood pressures, FBG=fasting blood glucose, free-T4=free thyroxine, HbA1c=glycosylated hemoglobin A1c, SBP= systolic blood pressure, TSH = thyroid-stimulating hormone, WC=waist circumference.

According to vitamin D values established by the Institute of Medicine, subjects with metabolic diseases group showed: 23% risk to bone health (9.42±3.04), 45% risk of insufficiency/ deficiency (17.05 ±2.12) and 32% had sufficient levels (26.34 ± 6.74) whereas HS group showed significantly higher values than metabolic diseases group (37.25 ±7.72).

Patients studied included: T2D (23.70%); hypothyroidism (23.70%); T2D + hypothyroidism (21.96%); EW (17.92%); and HS (12.72%). Therefore, the analysis of variance test was performed to study the differences between them (Table 2). Significant differences (P value <0.05) were only found for weight, height, BMI, SBP, FBG, and HbA1c. In particular, patients with T2D had higher SBP, FBG, and HbA1c compared to HS, hypothyroidism, and EW groups. On the contrary, T2D showed higher BMI compared to HS group. Patients with hypothyroidism showed significantly higher FBG and HbA1c compared to subjects with T2D + hypothyroidism; *high BMI and HbA1c in respect to HS and EW group, respectively.* Subjects with T2D + hypothyroidism had significant differences in SBP, FBG, and HbA1c compared to HS and EW groups. In addition, patients with T2D + hypothyroidism showed higher BMI compared to HS. Finally, EW exhibited significant differences in BMI and HbA1c respect to HS. There were no significant differences of 25-OHD levels between the groups with metabolic diseases, and there were only differences with HS.

**Table 2:** Levels of metabolic disorders parameters.

	T2D (n = 41)	Hypothyroidism (n=41)	T2D+ Hypothyroidism (n=38)	EW (n = 31)	HS (n=22)	*P*
Weight (kg)	77.78 ± 19.19	74.05 ± 13.86	80.73 ± 15.20^*^	78.90 ± 17.06	68.30 ± 8.44^*^	.003
Height (m)	1.65±0.08	1.60±0.06^†^	1.62±0.09^*^	1.65±0.07	1.69 ± 0.09^*,†^	.001
BMI (kg/m^2^)	28.28 ± 5.29^‡^	28.81 ±5.51^†^	30.68±4.83^*^	28.83±4.71^§^	23.73 ± 0.75^*,†,‡,§^	.0001
WC (cm)	94.60±16.77	88.61 ±15.61	93.43±13.30	87.15±17.97	84.00±9.53	.072
SBP (mm Hg)	133.55 ± 17.62^‡,§,‖,††^	120.94 ± 13.12^§,††^	130.36 ± 15.09^*,¶^	118.37 ± 8.85^‖,¶^	116.82±4.77^*,‡^	.0001
DBP (mm Hg)	68.81 ±8.99	65.65±8.37	69.57±7.84	70.84±10.73	70.00±6.90	.212
FBG (mg/dL)	131.16 ± 39.76^‡,§,||,††^	86.42 ± 10.49^§,#,††^	114.64 ± 34.84^*,¶,#^	87.76±9.00^‖,¶^	84.00±6.65^*,‡^	.0001
HbA1c (%)	7.68 ± 1.39^‡,§,‖,††^	5.17±0.25^§,#,**,††^	6.92 ± 1.03^*,¶,#^	5.64 ± 0.38^§,‖,¶,**^	5.25 ± 0.30^*,‡,§^	.0001
TSH (uUI/mL)	1.93±0.70	2.04±1.72	2.24±1.65	2.38±1.09	1.82±0.59	.304
Free-T4 (ng/dL)	1.28±0.27	2.34±2.40	1.81 ±2.04	1.64±1.11	1.51 ± 0.57	.330
Ca (mg/dL)	8.82±0.78	9.07±0.72	8.95±0.51	9.13±0.43	9.11 ± 0.66	.379
25-OHD (ng/mL)	18.50±6.72^‡^	19.42±9.48^†^	18.25±8.32^*^	19.56±4.54^§^	37.25 ± 7.72^*,†,‡,§^	.0001

One-way analysis of variance (ANOVA) test (significant *P*< .05).

25-OHD=25-OH vitamin D, BMI=body mass index, Ca=calcium, DBP=diastolic blood pressures, EW=excess weight, FBG=fasting blood glucose, free-T4=free thyroxine, HbA1c=glycosylated hemoglobin A1c, HS = healthy subjects, SBP=systolic blood pressure, T2D=type 2 diabetes, TSH = thyroid-stimulating hormone, WC=waist circumference.

* Post-hoc analysis revealed significant difference between HS and T2D+ hypothyroidism.

† Post-hoc analysis revealed significant difference between HS and hypothyroidism.

‡ Post-hoc analysis revealed significant difference between HS and T2D.

§ Post-hoc analysis revealed significant difference between HS and EW.

‖ Post-hoc analysis revealed significant difference between T2D and EW

¶ Post-hoc analysis revealed significant difference between T2D+ hypothyroidism and EW.

# Post-hoc analysis revealed significant difference between hypothyroidism and T2D+ hypothyroidism.

** Post-hoc analysis revealed significant difference between hypothyroidism and EW.

†† Post-hoc analysis revealed significant difference between T2D and hypothyroidism.

As shown in Table 3, in patients with metabolic disorders 25-OHD was inversely correlated with BMI, SBP, FBG, and HbA1c (P< .05). Although there is a significant difference, the association *r* is not strong. In contrast, 25-OHD did not show correlation with the rest of the studied parameters.

**Table 3:** Correlation between 25-OHD vitamin D levels and metabolic disorders parameters.

	25-OHD (ng/mL) levels	
	r	P
Weight (kg)	–0.109	.215
Height (m)	0.127	.148
BMI (kg/m^2^)	–0.194	.026
WC (cm)	–0.073	.406
SBP (mm Hg)	–0.283	.001
DBP (mm Hg)	–0.035	.694
FBG (mg/dL)	–0.284	.001
HbA1c (%)	–0.247	.004
TSH (uUI/mL)	–0.107	.222
Free–T4 (ng/dL)	–0.036	.682
Ca (mg/dL)	0.138	.116

Pearson correlation coefficient was applied. Significant *P<* .05.

BMI = body mass index, DBP=diastolic blood pressures, HbA1c = hemoglobin A1c, 25-OHD = 25-OH vitamin D, TSH = thyroid-stimulating hormone, SBP=systolic blood pressure, WC = waist circumference.

## Discussion

Vitamin D has multiple biological effects, such as calcium and phosphorus metabolism regulation and different cellular processes. Recent studies have also shown that vitamin D is closely involved in cardiovascular disease, diabetes, cancer, and others.^17^ It is, however, not common to evaluate vitamin D levels during the follow-up of these diseases because of its high costs. In this research we investigated the prevalence of *vitamin D deficiency* in subjects with common metabolic *disorders* in daily clinical practice and its relationship with other variables (anthropometric parameters, BP, glycemic status, thyroid profile, and calcium).

According to the bibliographic research, it is interesting to highlight the limited information on vitamin D levels in individuals with metabolic disorders in our region. They mostly describe vitamin D *status* in healthy adult populations at risk for diseases related to calcium and phosphorus metabolism.^18,19^This *study showed that about a quarter of patients with metabolic disorders had optimal vitamin D values. While a majority were vitamin D deficient or had risk to suffer deficiency. The optimal levels of these patients were, however, significantly lower than the healthy population. Similar results have been reported in other regions worldwide.*^20^

It has been reported that vitamin D deficiency and EW are associated.^21^ Although there is insufficient evidence to conclude whether vitamin D deficiency represents a cause or a consequence of EW. Several mechanisms have been suggested to explain this association. Volumetric dilution is the most acceptable: in overweight people, vitamin D is redistributed in a larger volume, resulting in significantly lower serum concentrations. Moreover, vitamin D is fat soluble and would accumulate in adipose tissue, which leads to lower plasma levels of vitamin D in overweight subjects. Other authors have reported impaired hepatic 25-hydroxylation as a possible mechanism for vitamin D deficiency in obese subjects with nonalcoholic fatty liver disease (one of the most common chronic complications in obesity). In addition, decreased gene expression of vitamin D metabolizing enzymes in patients with obesity could mean a deficit of the bioactive form of vitamin D and a reduction in its effect on the body. Recently, some studies support that vitamin D may be involved in the pathogenesis of obesity, rather than just being a consequence, due to their role in lipogenesis.^22^

This study found that all patients *with metabolic disorders* had higher mean BMI values compared to the HS group and kept a negative correlation with low vitamin D levels. Our results are consistent with those from Alloubani et al^23^ who studied *vitamin Ddeficiencies* in 350 Saudi Arabian participants with diabetes and/or obesity. *Delle Monache et al*^24^*demonstrated that BMI showed the highest significant inverse correlation with serum 25-OHD values, independently from season and age; therefore, they suggest it as a good predictor ofvitamin D status.* Moreover, two recent studies assessing hypothyroid patients and hypothyroid patients with T2D, that presented high BMI, also had low levels of vitamin D in comparison with its level in age-, weight-, and BMI-matched individuals without hypothyroidism and T2D.^25,26^ This may be associated with vitamin D role as an immunomodulating agent. Different immune system cells like antigenpresenting cells, B and T lymphocytes express vitamin D receptors and bind to it promote a tolerogenic immune status. For this reason, it has been suggested that lower plasma vitamin D levels would be associated with autoimmune diseases such as autoimmune hypothyroidism. Furthermore, in animal models, vitamin D has been shown to attenuate TSH-stimulated iodide uptake.^27^

In recent years our understanding of vitamin D effects on multiple organs apart from bone homeostasis has improved. High BP is considered a silent chronic disease that is linked to metabolic disorders such as diabetes and obesity. Animal model trials and clinical studies suggest that vitamin D deficiency is closely related to high BP.^28^ Nonetheless, these results are inconsistent and its role outside bone metabolism remains controversial.^29,30^

In the present study, SBP was inversely correlated with vitamin D plasma concentrations in patients with T2D. Similar findings were reported in a study of 116 patients from Brazil with T2D and hypertension, which showed a correlation between serum vitamin D levels and SBP.^31^ Moreover, recent studies in diabetics adults with hypothyroidism found that deficient vitamin D status were correlated with increased SBP.^25^ These results could be explained by the antihypertensive effect of vitamin D related to Renin-Angiotensin-Aldosterone System.^32^

A relevant impact of vitamin D has been suggested on insulin action and affects many pathways, which might be important for blood glucose regulation, as insulin synthesis and secretion. The mechanisms through vitamin D acts on insulin are direct and indirect. The direct effect of vitamin D would be due to the binding of the active form 1,25-OHD to the vitamin D receptor on pancreatic ß cells modulating insulin synthesis and secretion. On the contrary, indirect effects on the secretory function of cells appear to be mediated by alterations in calcium flux across the cell membrane.^33^ Furthermore, vitamin D appears to have impacts on proinflammatory cytokines, a significant element in the development of metabolic diseases *and insulin sensitivity, potentially linking insulin resistance and obesity. Vitamin D could interfere with cytokine generation by binding to the promoter site of a number of cytokine genes (modifying gene expression).*^34^

In this study results revealed that FBG and HbA1c were increased in all individuals with metabolic disorders and it correlated negatively with low vitamin D concentration. Indeed, glycemic control degree in patients with different pathologies in several studies showed a negative correlation between poor glycemic control and low vitamin D levels.^34–36^

The relevant finding of this research was to provide information on plasma vitamin D levels in patients with metabolic disorders in the northern region of Argentina, without any information available. Also, only subjects with an adequate nutritional intake of vitamin D-rich foods and frequent exposure to sunlight (a minimum of 15 min, 3 times per week) were included, so our results suggest an association between having some type of metabolic disorder and low vitamin D concentrations. In fact, suboptimal levels (<20 ng/mL) were found in the majority of patients. So we suggest that when diagnosing patients with metabolic disorders, vitamin D levels can be evaluated and proper treatment is administered, if any deficiency is seen.

This research has some limitations: it is a cross-sectional design that only allows association but no causality, and it is a preliminary study with a relatively small sample size. On the contrary, the experimental design used did not allow us to determine whether low vitamin D concentrations are a cause or consequence of pre-existing diseases, whether it could contribute to the development of associated complications, or whether optimal plasma vitamin D values would benefit the management of these individuals (reducing BMI, improving glycemic control, improving insulin sensitivity, and regulating BP). Finally, our study does not allow us to predict if subjects with metabolic disorders and adequate vitamin D values, but significantly lower than HS, will be at risk for vitamin D deficiency in the future.
